# Advances in saRNA Vaccine Research against Emerging/Re-Emerging Viruses

**DOI:** 10.3390/vaccines11071142

**Published:** 2023-06-24

**Authors:** Yalan Liu, Yuncheng Li, Qinxue Hu

**Affiliations:** 1 State Key Laboratory of Virology, Wuhan Institute of Virology, Center for Biosafety Mega-Science, Chinese Academy of Sciences, Wuhan 430071, China; 18839785259@163.com; 2 Hubei Jiangxia Laboratory, Wuhan 430200, China; 3 Savaid Medical School, University of Chinese Academy of Sciences, Beijing 101408, China; 4 Institute for Infection and Immunity, St George’s, University of London, London SW17 0RE, UK

**Keywords:** vaccine, self-amplifying RNA (saRNA), emerging/re-emerging virus

## Abstract

Although conventional vaccine approaches have proven to be successful in preventing infectious diseases in past decades, for vaccine development against emerging/re-emerging viruses, one of the main challenges is rapid response in terms of design and manufacture. mRNA vaccines can be designed and produced within days, representing a powerful approach for developing vaccines. Furthermore, mRNA vaccines can be scaled up and may not have the risk of integration. mRNA vaccines are roughly divided into non-replicating mRNA vaccines and self-amplifying RNA (saRNA) vaccines. In this review, we provide an overview of saRNA vaccines, and discuss future directions and challenges in advancing this promising vaccine platform to combat emerging/re-emerging viruses.

## 1. Introduction

Epidemics characterized by high rates of morbidity and mortality have always existed alongside human beings. There are numerous examples of major epidemics in past centuries. We have witnessed an unprecedented rise in the emergence of new infectious diseases and the re-emergence of old ones in recent decades. The latest outbreak of the coronavirus disease 2019 (COVID-19) pandemic caused by severe acute respiratory syndrome coronavirus 2 (SARS-CoV-2) [1] affects people worldwide and continues to severely influence peoples’ social life and economic activities. Although conventional vaccine approaches have been successful in preventing several infectious diseases in recent decades, for most vaccines against emerging viruses, the main challenge is the need for a rapid response and large-scale development. The success of mRNA vaccines in preventing COVID-19 demonstrates a promising approach for designing vaccines against other emerging/re-emerging viruses. mRNA vaccines can be quickly produced within days after obtaining a nucleic acid sequence of the virus immunogen, and are capable of inducing both humoral and cell-mediated immunity. Like DNA vaccines, mRNA vaccines are easy to scale up, and may not have the risk of integration into the genome of the host. mRNA vaccination has also been reported to result in a balanced IgG1/IgG2a response [2,3], which plays an important role in curtailing the severity of emerging virus outbreaks. mRNA vaccines can be roughly divided into two categories based on whether they have the capability to self-replicate in vivo: non-replicating mRNA (conventional linear RNA and newly reported circular RNA [4]) vaccines and self-amplifying RNA (saRNA) vaccines. Compared to a non-replicating mRNA vaccine, a similar protein expression level of an immunogen and equivalent protection efficacy against a virus could be achieved by a saRNA vaccine at a lower dose [5]. The use of a lower dosage of saRNA would minimize the usage of delivery materials, such as cationic liposomes, facilitating the control of the cost and potential side effects. Furthermore, the expression of a saRNA vaccine in vivo could last 1–2 months [3,6,7], making it feasible to achieve sufficient protection with a single immunization [8,9,10]. In this review, we focus on saRNA vaccines by summarizing the advances in this field and discussing the perspectives and challenges of saRNA vaccines to combat emerging/re-emerging viruses.

## 2. Structural Characterization of saRNAs

Although a conventional mRNA is relatively simple and straightforward to transcribe in vitro, a large dose of the mRNA or repeated immunization procedures may be needed in order to elicit sufficient immune responses. As an alternative, saRNA vaccines have been under development to address such limitations. Structurally, a saRNA vaccine encodes a replicon which functions as viral replication machinery to amplify intracellular RNAs, and also has the components of conventional mRNA vaccines [11] (reviewed in reference [12]). To date, genetically engineered replicons have been commonly derived from the genomes of single-stranded RNA viruses, of which most were positive-sense alphaviruses, such as Venezuelan equine encephalitis virus (VEEV), Sindbis virus (SINV), and Semliki Forest virus (SFV) [7]. In some cases, replicons from other viruses have also been used, including classical swine fever virus (CSFV) [13,14,15], tick-borne encephalitis virus (TBEV) [16,17], and norovirus [18]. The replicase genes of these viruses encode an RNA-dependent RNA polymerase (RdRp) complex which amplifies the RNA of the immunogen. Therefore, saRNA vaccines can be delivered at a lower dose than conventional mRNA vaccines to achieve similar levels of immune responses, and in theory reduce the frequency or necessity of booster administrations [5,10]. Although saRNA contains non-structural proteins (nsP1-4), immune responses against the nsPs have not been observed upon subsequent boosts with a viral replicon particle vaccine in animal studies [19], indicating that saRNA is safe for clinical application.

## 3. Design Strategies for saRNAs

To improve the stability and application performance, mRNA vaccine optimizations have been focused on several aspects such as purification of in vitro transcribed (IVT) mRNA [20], optimization of mRNA sequences [21,22,23,24,25,26,27,28,29,30], and formulation of mRNA with various carrier molecules [31,32,33,34,35]. For instance, various versions of 5’ cap structure [23,24,25,26], and an optimal length of the poly(A) tail [27] were assessed. The replacement of rare codons with frequently used synonymous codons [28], enrichment of G:C content [29,30], triazole-modification of DNA template [36], and incorporation of pseudouridine/N(1)-methylpseudouridine into mRNA [37,38] have also been tested. In addition, the alteration of 5’ and 3’ UTRs or the insertion of a fixed-length sequence of poly(A) tail into DNA templates have been investigated [39].

In addition to the various design strategies discussed above, which can be applied to both conventional mRNAs and saRNAs, there are other unique designs applicable to saRNAs. It has been reported that an RNA-based adjuvant can enhance virus-specific vaccine responses [40], while self-adjuvanted mRNA vaccines induced local innate immune responses [41,42]. saRNAs can induce interferon (IFN)-mediated antiviral response [43,44], whereas innate recognition of mRNAs upregulates the expression and activation of protein kinase R (PKR) and 2’-5’-oligoadenylate synthetase (OAS), which leads to inhibition of translation [45]. To reduce excessive immune stimulation, saRNA constructs which cis-encode innate immunity-inhibiting proteins (IIPs) have been shown to effectively abate the nonlinear dose dependency and enhance immunogenicity [46]. The addition of elements that regulate (induce or restrict) the innate immunity is another direction of saRNA optimization.

**Figure 1:**
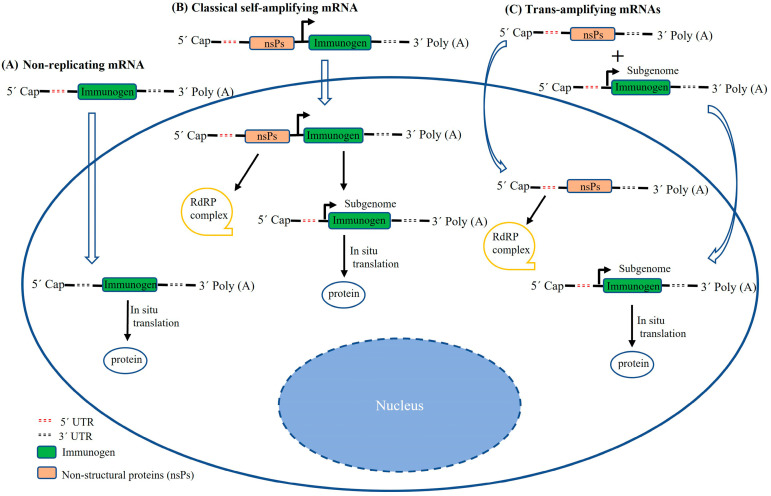
Schematic illustration of different RNA transcripts delivered to a target mammalian cell. (**A**) Delivery of non-replicating mRNA to the cytoplasm. (**B**) Delivery of classical saRNA to the cytoplasm. Following in situ translation, the non-structural proteins (nsPs) form an RdRP complex which amplifies immunogen-encoding transcripts. (**C**) Two different transcripts (trans-amplifying mRNAs) are co-delivered to the cytoplasm, which achieve a similar effect to self-amplifying mRNAs.

Classical saRNAs are larger (~10 kb) than conventional mRNAs. The yield of saRNAs produced in vitro is much lower than that of conventional mRNAs. Recently, Beissert et al. developed a novel trans-amplifying RNA (taRNA) system [47]. This taRNA system consists of two vectors: vector 1 encodes the viral immunogen, and vector 2 contains an alphaviral replicon which produces a replicase complex and is capable of amplifying the transcript of vector 1 in trans (Figure 1). Compared to classical saRNAs, the taRNA system was shown to have higher translational efficiency and less interference with cellular translation. Influenza hemagglutinin antigen-encoding RNA based on this taRNA system has been proven to robustly enhance viral immunogen expression and induce protective immune response against live virus challenge with a dose as low as 50 ng in mice [47]. Although whether the two elements of taRNAs are transferred into the same cell has not yet been confirmed, the universal applicability of this taRNA system is worthy of further exploration.

## 4. Administration Routes and Delivery Systems for saRNAs

Despite the use of IVT mRNA in animals as early as 1990 [48], the promising results did not lead to a successful development of mRNA therapeutics, largely because mRNA can easily be degraded by RNase and has high innate immunogenicity, as well as the lack of an efficient in vivo delivery system. It is known that antigen expression is positively associated with the number of mRNA transcripts successfully delivered during vaccination. A number of delivery systems have been demonstrated to have various efficacies through intradermal [49], intrasplenic [10], subcutaneous [14], intravenous [49,50], and even intranasal [51] routes of administration. Because the intramuscular (IM) route is a suitable route for the delivery of mRNA to lymph node DCs [52], most saRNA vaccines have been investigated via IM injection in mice, macaques, and humans. 

Delivery of saRNAs in vivo is a complex multistep process and there is a need to avoid RNase-mediated degradation and clearance. In general, mRNA delivery can be roughly divided into viral delivery systems and nonviral delivery systems. The viral delivery systems deliver saRNAs via viral replicon particles (VRPs) in which the saRNAs are packaged. In the simplest form, the structural protein genes of an alphavirus or other RNA viruses were replaced with a heterologous gene encoding the immunogen. The transformed RNA (so-called replicon) can amplify its own heterologous gene. If these replicons are introduced into helper cells in which the structural genes are expressed in trans, VRPs containing the protein and lipid structure of wild-type viruses and transformed RNAs are produced. VRPs are single-cycle infectious particles which can be inoculated in animals. VRPs express the heterologous gene but are incapable of producing viral particles or spreading cell-to-cell due to the lack of structural protein genes in the saRNA. Although VRP-based vaccine candidates have been tested in a variety of small animals, non-human primate models and humans [53,54], anti-vector neutralizing immunity was also observed against VRPs [55,56]. The nonviral delivery systems deliver saRNAs in one of the following ways: naked [57]; through a gene gun [17]; by electroporation [2]; or formulated in lipid nanoparticles (LNPs) [3], nanostructured lipid carrier (NLC) [10], cationic nanoemulsion (CNE) [6], pABOL [33], or neutral lipopolyplex (LPP) [35]. Although several reported saRNA vaccines against respiratory syncytial virus (RSV), Louping-ill virus (LIV), or influenza viruses delivered nakedly had obviously protective effects [57,58,59], the incorporation of RNAs into particles which can protect RNAs from enzymatic degradation and improve delivery efficiency is a high priority. The reported delivery approaches employed for saRNA delivery are listed in Table 1.

The first LNP-formulated RNA was developed in 2018, followed by a clinical safety assessment of the LNP-formulated RNA [31]. Currently, LNPs are the most applied saRNA delivery system in preclinical and clinical studies, and various LNP platforms have been developed [65]. LNPs often consist of four components: ionizable cationic lipid, lipid-linked polyethylene glycol (PEG), cholesterol, and naturally occurring phospholipids. A number of studies have demonstrated the in vivo efficiency of siRNAs or conventional mRNAs delivery by LNPs. LNPs as a tool for in vivo delivery of saRNAs were first reported one decade ago [3], and since then increasing numbers of researchers have been choosing LNPs as a tool for saRNA delivery. For instance, Englezou et al. identified the form of the cationic lipid molecule, providing the most efficient lipoplexing that facilitates saRNA delivery in DCs both in vitro and in vivo [14]. Blakney et al. established the formulation of saRNAs on the surface of cationic LNPs as an alternative to the paradigm of encapsulating RNAs [66], while Goswami et al. used mannosylation of LNPs (MLNP) to achieve a faster immune response to saRNAs independent of the delivery route [34].

Although LNP formulation has been increasingly used in various saRNA vaccinations which showed potential capacity against the targeted viruses [67], it is difficult to be stored in large quantities and for long periods of time [68]. Erasmus et al. developed a novel delivery system, a highly stable nanostructured lipid carrier (NLC), to package saRNAs [10]. Technically, RNA needs to be encapsulated into LNP first and then stored or transported under suitable conditions for vaccination. Unlike LNP, NLC can be manufactured and stored separately from mRNAs, and mixed prior to administration. Furthermore, specific physicochemical modifications to the NLC can change the intensity of the immune responses.

CNE has been proven to be an effective saRNA delivery tool in a number of animal models (mice [6,69,70], rats [51], rabbits [6], ferrets [69], and rhesus macaques [6,63]). Brito et al. developed a delivery system in which CNE contained the cationic lipid DOTAP (1,2-dioleoyl-sn-glycero-3-phosphocholine) and co-vaccinated with the licensed MF59 (Novartis) adjuvant [6]. Although delivery of siRNA or pDNA by cationic lipids has been reported to be poorly tolerated [71], data from Brito et al. demonstrated that a CNE-delivered saRNA vaccine was well tolerated and displayed increased immunogenicity and efficacy [6]. One study which compared four different cationic platforms revealed that DOTAP polymeric nanoparticles appeared to be the most potent in triggering humoral and cellular immunity among candidates in vivo [32].

Considering the structure differences of siRNAs, conventional mRNAs and saRNAs, Blakney et al. developed a high-molecular-weight, bio-reducible, linear cationic polymer called “pABOL” to deliver saRNAs. pABOL can enhance the expression level of immunogens and the cellular uptake of pABOL-delivered saRNAs via both intramuscular and intradermal injection. The assessment of the immunogenicity and protective capacity of saRNAs delivered by pABOL indicated that pABOL-delivered saRNA encoding hemagglutinin (HA) induced high HA-neutralizing antibodies and could protect mice against influenza virus challenge [33]. Because pABOL can be synthesized on a large scale and produced easily, it has certain advantages for saRNA delivery.

The major limitations with large and complex saRNA vaccines are RNase sensitivity and inefficient translation in dendritic cells (DCs). Demoulins et al. improved the polyplex formulation and demonstrated that fine-tuning of the polyplex structure is essential for ensuring efficacious translation [15]. Perche et al. found that it was able to encapsulate RNAs into neutral lipopolyplexes (LPPs) consisting of cationic polymer and anionic liposomes. LPPs were stable in vitro and successfully delivered conventional RNAs and saRNAs to DCs. Administration of LPP-saRNAs also led to an adaptive immune response [35].

Cationic lipids with higher doses or which are incompletely complexed can be toxic [71]. Furthermore, the immunogenicity of cationic lipids raises safety concerns [72], and for instance, lipid-complexed mRNA can induce IFN production, which limits the efficacy of mRNA-based vaccines [73]. Chahal et al. developed a dendrimer nanoparticle vaccine platform based on MDNPs composed of ionizable delivery materials and lipid-anchored PEG [64]. As saRNA delivered through MDNP delivery technology does not generate a systemic increase in inflammatory cytokine production, it can avoid the influence of early IFN responses which affect alphavirus replication, and thus minimizes the dose of vaccine being used [74,75] and prevents antivector immunity [76]. Such an approach is capable of eliciting both CD8^+^ T-cell and antibody responses, and induces protective immunity against a broad spectrum of lethal pathogen challenges, including H1N1 influenza virus, Toxoplasma gondii, and Ebola virus with a single dose [64].

## 5. saRNA Vaccines against Emerging/Re-Emerging Viruses

Global public health has been seriously threatened by the emergence/re-emergence of viral infectious diseases. Such diseases are caused at least in part due to the following reasons: highly increased global connectivity via air travel and international trade links, and the loss of the natural living environments of wild animals owing to the serious environmental destruction caused by human beings, such as deforestation and climate change, which all serve to co-localize humans with animal reservoirs and alter the habitat of vector species, thus facilitating the transmission of viruses between species [77,78]. As shown in Table 2, saRNA vaccines have been applied for various emerging or re-emerging viruses, such as SARS-CoV-2, HIV-1, influenza viruses, rabies virus, Zika virus (ZIKV), RSV, and Ebola virus (EBOV). In particular, we discussed saRNA vaccines which have been developed against the infection of these viruses.

### 5.1. SARS-CoV-2

SARS-CoV-2 infection and the resulting COVID-19 have deeply affected people and economy globally. Since the outbreak, a number of vaccines have been rapidly developed against the virus and have achieved success in controlling the diseases. However, the emergence of multiple variants of SARS-CoV-2, particularly those with the potential to escape vaccine-induced immunity is compromising the protective efficacy of the vaccines being used [90]. To meet the global demand and to combat the emergence of new SARS-CoV-2 variants, more saRNA vaccines have been developed and assessed in mice [9,82,83,84,85], hamsters [81,82], non-human primates [84,85], and humans [62,79,80].

So far, two saRNA vaccines have undergone clinical studies. Both SARS-CoV-2 saRNA vaccines were encapsulated in LNP and administered as intramuscular (IM) injections. One SARS-CoV-2 saRNA vaccine was developed by COVAC 1 Study Team and has completed phase I and phase 2a trials. The phase I study enrolled 192 healthy individuals with no history or serological evidence of COVID-19, aged 18–45 years. Participants were administered two IM doses 4 weeks apart. Data in phase I study demonstrated that the vaccine was well tolerated with no serious adverse events (AEs) related to vaccination. Seroconversion was related to dose, ranging from 8% (3/39; 0.1 μg) to 61% (14/23; 10.0 μg) in ELISA and 46% (18/39; 0.3 μg) to 87% (20/23; 5.0 μg and 10.0 μg) in immunoblot assay. Although the saRNA vaccine failed to induce 100% seroconversion, it was safe for clinical development, and immunogenic at low doses [62]. The latest phase 2a trial enrolled a larger population (216 healthy individuals) with a wider age range (18–75 years), stable co-morbidities, and previous immunity to SARS-CoV-2 to expand study of the safety and immunogenicity of this saRNA. Participants received two IM injections with a longer interval (a median of 14 weeks compared to 4 weeks) and doses were 1 μg followed by 10 μg. The vaccine was well tolerated in adults with fewer AEs with increasing age. Seroconversion rates were significantly higher than those previously reported in phase I trial. Data in phase 2a supported its use in a wider cohort, including older people, people with co-morbidities, and with previous immunity to SARS-CoV-2, raising no safety concerns [80]. Another SARS-CoV-2 saRNA vaccine, ARCT-021, was developed by Jenny G. Low et al., and has undergone a phase I/II trial to assess its safety, tolerability, and immunogenicity at different dose levels [79]. ARCT-021 has been proven to elicit strong Th1-predominant humoral and cellular immune responses with a single dose in ACE2-transgenic mice in the preclinical study [9]. The clinical trial participants were healthy young (21–55 years) and older (56–80 years) adults. ARCT-021 was administered with one injection in the phase I trial and two same-dose injections with 28 days apart in the phase II trial. It was well tolerated up to one 7.5 μg dose and two 5.0 μg doses. Local solicited AEs were more common in ARCT-021-vaccinated recipients, while the difference of systemic solicited AEs existed in ARCT-021 and placebo recipients was not obvious (62.8% vs. 46.4%). Seroconversion rate for anti-S IgG was 100% in all cohorts, except for the 1 μg one-dose in younger adults and the 7.5 μg one-dose in older adults. The saRNA construct of ARCT-021 contained an unmodified S gene in the preclinical study [9] and phase I/II trials [79]. In addition, a modified S gene saRNA construct (ARCT-154) has successfully progressed through immunogenicity trials to a phase III clinical trial in Vietnam, which showed 95.3% efficacy against severe COVID-19 (Clinicaltrials.gov identifier: NCT04480957).

### 5.2. HIV-1

Although antiretroviral therapy (ART) has transformed HIV-1 infection from a fatal disease to a chronic disease that can be controlled by drugs, HIV-1 infection remains a pandemic with no cure and no vaccine available [91,92,93]. As early as in 1997, Berglund et al. used a recombinant Semliki Forest virus (SFV) RNA vector encoding the envelope protein gp160 of HIV-1 IIIB to immunize cynomolgus macaques, followed by challenge with chimeric simian–human immunodeficiency viruses (SHIVs) to evaluate the SFV-based RNA vaccine. Three out of four vaccinated monkeys had no demonstrable viral antigenemia or low viral load as opposed to one of the four naive control animals [94]. In addition, several SFV- or VEEV-based vaccines were reported, which delivered HIV-1 envelope glycoprotein (Env) immunogens by the viral delivery systems. Although VRPs expressing HIV-1 immunogens all elicited potent immune responses [94,95,96,97,98], only Wecker et al. conducted a clinical experiment and reported the results of the phase I trial of their HIV-1 vaccine AVX101, a recombinant VRP vaccine expressing a subtype C gag gene, modified to express nonmyristoylated Gag. However, anti-vector neutralizing immunity was observed against VRPs and the immune responses in humans were not as good as those in the preclinical studies. Only low levels of binding antibodies and T-cell responses were seen at the highest doses [99]. With the advancement of technology, other delivery systems, such as LNP [3] and CNE [6], have been developed to replace VRP. Aldon et al. evaluated the potential of VEEV-based saRNAs encoding HIV-1 Env trimers in polyplex (PLX) formulations to induce potent immune responses in mice. This polymer-formulated saRNA encoding the membrane-bound Env also induced high IgG response in larger animal models, including guinea pigs, rabbits, and macaques [61]. In the latest research, HIV-1 saRNAs were formulated with lipid inorganic nanoparticles (LIONs), which can enhance vaccine stability, delivery, and immunogenicity to evaluate immunogenicity in pregnant rabbits [100]. So far, no HIV-1 saRNA vaccines have been evaluated in clinical trials.

### 5.3. Influenza Viruses

Influenza viruses are pathogens with pandemic potential, having caused three pandemics in the 20th century. The prevention and control of influenza virus infection remains a huge challenge, as it is hard to predict the pandemics. The vaccine formulation needs to be constantly updated against prevalently circulating virus subtypes before each influenza season because of the high mutation rate of influenza viruses. Although various influenza vaccine approaches, such as subunit vaccines, inactivated vaccines, and live-attenuated vaccines are available, saRNA vaccines may offer a quick response to seasonal epidemics and pandemics. For example, a saRNA vaccine platform showed its rapid response capabilities against the outbreak of H7N9 influenza in China. Seven days after the announcement of the outbreak, a saRNA vaccine encoding influenza H7 HA antigen against H7N9 (A/Shanghai/2/2013) was developed and subsequently tested in a small animal model [67].

So far, saRNA vaccines which can be quickly produced and flexibly modified have been introduced for protection against H1N1 [5,33,34,35,47,64,69,87,88], H3N2 [5,87], and H7N9 [67,69]. Although the preclinical data from animal models indicated that saRNA vaccines have potential for clinical application, no clinical trials have been initiated with influenza saRNA vaccines. The immune responses and protection efficacy of candidate influenza saRNA vaccines in humans remain to be investigated.

### 5.4. Other Viruses

In addition to the viruses mentioned above, saRNA technology has also been applied to fight against other emerging/re-emerging viruses, such as rabies virus, ZIKV, RSV, and EBOV. To date, only one, a rabies saRNA vaccine (RG SAM) developed by GSK, has undergone a clinical trial (ClinicalTrials.gov Identifier: NCT04062669) to evaluate its safety, reactogenicity, and immunogenicity. The vaccine consists of an engineered replication-deficient alphavirus genome and the gene of the full-length rabies glycoprotein G in combination with the delivery system CNE. In the phase I study, 18–40-year-old healthy adults were administered with RG SAM intramuscularly on a 0-, 2-, and 6-month schedule. The data of the clinical trial have not been published yet. The local tolerance, potential systemic toxicity, and biodistribution of RG SAM were evaluated in a rat model [51], showing that the rabies saRNA vaccine was well tolerated and supporting the clinical development program. GSK also performed a nonclinical safety assessment of LNP-and CNE-based saRNA vaccines in rats [89]. The saRNA vaccines, administered as two doses 2 weeks apart, had acceptable safety profiles in rats with respect to clinical signs, blood biochemistry, and macroscopic and microscopic pathology. In addition, a set of cationic formulations for rabies saRNA vaccine delivery were investigated, revealing that saRNA encapsulating DOTAP polymeric nanoparticles, DOTAP liposomes, or DDA liposomes induced the highest antigen expression in vitro, and among them, DOTAP polymeric nanoparticles were the most potent in triggering humoral and cellular immunity in vivo [32].

ZIKV is associated with an increased incidence of neurological complications, including Guillain-Barré syndrome [101] and fetal abnormalities [102]. There are no approved vaccines for ZIKV infection. Considering the threat of the ZIKV outbreak and the likelihood of its continuing transmission worldwide, developing a saRNA vaccine with rapid response may be critical. There have been three reported saRNA vaccine candidates against ZIKV, which all use the pre-membrane and envelope (prM-E) glycoproteins of ZIKV as the immunogens and have been developed and tested in preclinical studies [10,49,50,86]. Erasmus et al. demonstrated that a single dose as low as 10 ng of their saRNA vaccine delivered by NLC could completely protect mice against a lethal ZIKV challenge [10], while Chahal et al. developed a MDNP-based saRNA vaccine which elicited ZIKV E protein-specific IgG responses and protected mice from ZIKV infection after a single-dose immunization [50]. Furthermore, Zhong et al. designed and evaluated the immunogenicity and protection efficacy of a naked ZIKV saRNA vaccine in IFNAR1 knockout C57BL/6 mice (IFNAR1-/- mice) [49], and this unformulated saRNA vaccine elicited highly reproducible antibody titers in IFNAR1-/- mice and protected mice against ZIKV challenge. However, in wild-type (WT) C57BL6 mice, the vaccine elicited much lower and highly variable antibody titers, indicating that the elicited type I IFNs had a negative impact on the antibody titers.

RSV infection causes lower respiratory tract infection. The F protein of RSV is a conserved target for neutralizing antibody induction and vaccine development [103]. The first saRNA vaccine of RSV was developed as a recombinant Semliki Forest virus (rSFV) RNA-encoding RSV F protein. Unlike VRPs, the rSFV RNA vaccine did not contain viral structural components, which are often highly immunogenic. Due to the lack of a good delivery system at that time, mice were immunized with a naked rSFV RNA. In spite of this, significant levels of protection against RSV infection in mice were achieved [58]. In addition, Andrew J. Geall et al. developed a RSV saRNA vaccine, utilizing LNP as the delivery system. Compared with VRPs and pDNA, the efficiency of antigen production and immunogenicity of this LNP-saRNA vaccine in mice and cotton rats increased [3]. Subsequently, this team continued to develop a CNE delivery system to deliver RSV saRNA. The RSV CNE-delivered saRNA vaccine elicited potent immune responses in mice comparable to a viral delivery technology. They also demonstrated that saRNA delivered by a CNE was well tolerated and immunogenic in a variety of animal models, including mice, rats, rabbits, and nonhuman primates [6]. Nevertheless, so far, there is still no RSV saRNA vaccine being tested in clinical trials.

It is known that EBOV infection can cause severe clinical symptoms, including hemorrhagic fever and multiorgan failure, or more common symptoms such as fever, malaise, headache, diarrhea, and/or vomiting [104]. To date, one saRNA vaccine candidate for EBOV has been reported, which was a MDNP-delivered VEEV replicon RNA encoding the EBOV glycoprotein (GP). The vaccine candidate was shown to protect mice against lethal viral infection and was capable of eliciting both CD8(+) T-cell and antibody responses [64].

## 6. Challenges and Perspectives

Although there is a rapid increase in the use of saRNA vaccines in preclinical and clinical studies, a number of questions remain to be addressed before saRNA vaccines become widely applicable. Firstly, saRNA is too large. Although Beissert T. et al. developed a trans-amplifying RNA vaccine strategy by which the long saRNA was divided into two different and smaller transcripts [47], the bigger transcript which contained the viral replication machinery was still too large. Sequences of nsPs with higher catalytic efficiency and shorter length should be tested in future studies. Chromatographic separation of RNA is effective in purifying RNA molecules of up to about 4000–5000 bases, which is unlikely to fit for saRNA purification. Thus, there remains a need to purify large RNAs whose stability and biological activity are not disrupted, whereas large-scale chromatographic purification of large RNAs is a technical problem to be solved. Secondly, storage and transportation of saRNA vaccines is also a challenge because of the poor stability of RNAs. Studies on the stability of RNAs demonstrated that an intact and stable cap structure is necessary for transcription initiation and RNAs to be functional [105]. While using lyophilization to stabilize RNAs during storage was proven to be feasible [106,107], much more research will be required to develop straightforward and economical methods. Thirdly, most studies have proven that the saRNA vaccines tested so far are capable of producing potent and robust innate and adaptive immune responses in small animals, while the related research of antagonizing pathogenic challenge in big animals and humans is lacking except for SARS-CoV-2 saRNA vaccines. Due to inherent differences in innate immunity between different species, further research into immunogenic and well-tolerated saRNAs in big animals and humans is warranted in future. Fourthly, although various optimized delivery platforms have been developed, a form of refined pharmacokinetics in vivo has yet to be determined. Due to the lack of parallel comparisons, it is difficult to decipher which delivery strategy is the best. The evaluation of clinical efficacy and possible side effects are of equal importance and need to be elucidated. Furthermore, cationic lipids with highly efficient delivery capability also trigger toxic pro-apoptotic and pro-inflammatory responses [108,109]. Although ionizable lipids have been developed to overcome these safety concerns, more improved strategies should be considered in future research.

## Figures and Tables

**Table 1:** Delivery approaches for saRNA vaccines.

Delivery Approaches		Features	Advantages	Disadvantages	Examples of References
**Naked**		Formulated in buffer; direct injection	Simple; low cost	RNA susceptible to degradation by RNase	[57,58,59]
**Gene gun or electroporation**		Physical techniques; device-mediated	Safe; simple	Harmful to cells; low efficiency	[2,17]
**Viral delivery**		Viral replicon particles (VRPs)	Single-cycle infectious particles	High cost; anti-vector neutralizing immunity	[53,54]
**Nonviral delivery**	Liposomes	Lipid mixtures composed of DOPE and a cationic lipid (DOTAP or DDA)	Low toxicity and biocompatibility	Low encapsulation capacity	[60]
	Polymer polyethylenimine (PEI)	Cationic polymers being used to formulate nanoparticles	Low cost, high transfection efficiency, and high escape efficiency from intracellular bodies	High cation density can result in severe toxicity	[5,61]
	LNP	Composed of a complex amino lipid (either ionizable or non-ionizable), a phospholipid, cholesterol, a poly (ethylene glycol)-lipid conjugate, and the RNA	Protects RNA against degradation; assists in endocytosis and endosomal escape; markedly enhances the potency of the saRNA	High cost; repeated application can induce an immune response against polyethylene glycol; difficult to be stored in large quantities and for long periods of time; ionizable amino lipids have certain toxicity	[3,14,34,61,62]
	CNE	A mixture of an aqueous phase containing buffer and Tween 80 with an oil phase containing Span 85, DOTAP, and squalene	Effective; well tolerated for saRNA; enhances RNA delivery, and thereby substantially increases the potency of the vaccine; the duration and magnitude of immunogen expression are similar to the LNP delivery system	Limited to saRNA use	[6,51,63,64,65]
	Cationic polymer “pABOL”	Bioreducible, linear, cationic polymer; higher transfection efficiency and lower cytotoxicity compared to commercially available PEI	Less cytotoxic at higher molecular weights; enhances the expression level of immunogen and the cellular uptake; can be synthesized on a large scale and produced easily	Not described	[33]
	Neutral lipopolyplexes (LPPs)	Ternary complexes composed of a cationic polymer and mannosylated liposomes	Stable in vitro and can delivery RNAs to DCs; protects RNAs from degradation	Not described	[35]
	NLC	Composed of a hybrid liquid squalene and solid glyceryl trimyristate (Dynasan 114) core	Highly stable; can be manufactured and stored separately from RNAs; sufficient RNA-loading capacity; specific physicochemical modifications can change the intensity of the immune responses	Not described	[10]
	Modified dendrimer nanoparticle (MDNP)	Composed of ionizable dendrimer-based nanomaterial, a lipid-anchored PEG and the RNA	Stable; protects RNA payloads; free of infectious contaminants and virtually endotoxin-free; no systemic increase in inflammatory cytokine production	Not described	[64]

**Table 2:** saRNA vaccines against emerging/re-emerging viruses.

Viruses	Immunogen	Replicon	Species	Delivery System	Administration Route
SARS-CoV-2 [62]	Spike protein	VEEV	Human	LNP	IM
SARS-CoV-2 [79]	Spike protein	VEEV	Human	LNP	IM
SARS-CoV-2 [80]	Spike protein	VEEV	Human	LNP	IM
SARS-CoV-2 [81]	Spike protein	VEEV	Hamsters	LNP	IM
SARS-CoV-2 [82]	RBD and NP	VEEV	Mice/hamsters	LNP	IM
SARS-CoV-2 [9]	Spike protein	VEEV	Mice	LNP	IM
SARS-CoV-2 [83]	Spike protein	VEEV	Mice	LNP	IM
SARS-CoV-2 [84]	RBD and full-length spike protein	Unknown	Mice/rhesus macaques	LNP	IM
SARS-CoV-2 [85]	Spike protein	VEEV	Mice/pigtail macaques	LIONs	IM
SARS-CoV-2 [18]	Spike protein	Norovirus GI	Mice	LNP	Intranasal
HIV-1 [86]	Env	VEEV	Mice	LNP	IM
HIV-1 [60]	Gag/Pol mosaic	SFV	Mice	Polyplus Transfection	IM
HIV-1 [63]	Env	VEE–SINV	Rhesus macaques	CNE	IM
HIV-1 [61]	Native-like Env trimers	VEEV	Mice, guinea pigs, rabbits, macaques	PEI	IM
HIV-1 [2]	Env	VEE–SINV	Mice	Electroporation (naked)	IM
HIV-1 [6]	Env	VEE–SINV	Rabbits/rhesus macaques	CNE	IM
HIV-1 [3]	Env	VEE–SINV	Mice	LNP	IM
Influenza virus [5]	HA	Not described	Mice	PEI	IM
Influenza virus [67]	HA	Alphavirus replicon	Mice	LNP	IM
Influenza virus [69]	HA	VEE–SINV	Mice/ferrets	CNE	IM
Influenza virus [64]	HA	VEEV	Mice	MDNP	IV
Influenza virus [33]	HA	VEEV	Mice	pABOL	IM
Influenza virus [34]	HA	Not described	Mice	MLNP	IM/IV
Influenza virus [35]	HA	VEEV	Mice	LPP	IM
Influenza virus [47]	HA	Trans-amplifying	Mice	Naked	ID
Influenza virus [59]	NP	SFV	Mice	Naked	IM
Influenza virus [13]	HA/NP	CSFV	Mice/rabbits	Chitosan NGA	IM
Influenza virus [87]	M1/NP	VEE–SINV	Mice	LNP	IM
Influenza virus [88]	NP	VEE–SINV	Mice	LNP	IM
Influenza virus [15]	HA/NP	CSFV	Pigs	CPP PEI	IM
Influenza virus [14]	NP	CSFV	Mice	Cationic lipid	IH
Rabies virus [51]	Glycoprotein G	VEE–SINV	Rats	CNE	IM
Rabies virus [32]	Glycoprotein G	VEE–SINV	Mice	Liposome, nanoparticle, CNE	IM
Rabies virus [89]	Glycoprotein G	VEE–SINV	Rats	LNP/CNE	IM
ZIKV [49]	prM-E	VEEV	Mice	Electroporation (naked)	ID, IV.
ZIKV [10]	prM-E	VEEV	Mice	NLC	Intrasplenic
ZIKV [50]	prM-E	VEEV	Mice	MDNP	IV
RSV [6]	F glycoprotein	VEE–SINV	Mice	CNE	IM
RSV [3]	F glycoprotein	VEE–SINV	Mice/Rats	LNP	IM
RSV [58]	F glycoprotein	SFV	Mice	Naked	IM
Ebola virus [64]	Glycoprotein	VEEV	Mice	MDNP	IV
TBEV [16,17]	Capsid-null TBEV particles	TBEV	Mice	Gene gun	/
VEEV [70]	Glycoprotein	VEEV	Mice	CNE	IM
LIV [58]	prME	SFV	Mice	Naked	IM

LIV: Louping ill virus, RBD: receptor-binding domain, HA: haemagglutinin, NP: nucleocapsid protein, prM-E: pre-membrane and envelope glycoproteins, LIONs: lipid inorganic nanoparticles, IM: intramuscular injection, IV: intravenous injection, ID: intradermal injection, IH: subcutaneous injection.

## Data Availability

Not applicable.
